# Perception of Egocentric Distance during Gravitational Changes in Parabolic Flight

**DOI:** 10.1371/journal.pone.0159422

**Published:** 2016-07-27

**Authors:** Gilles Clément, Nuno Loureiro, Duarte Sousa, Andre Zandvliet

**Affiliations:** 1 Lyon Neuroscience Research Center, Bron, France; 2 Champalimaud Neuroscience Programme, Champalimaud Centre for the Unknown, Lisbon, Portugal; 3 International Space University, Strasbourg, France; 4 European Space Research and Technology Center, European Space Agency, Noordwijk, The Netherlands; University of Waterloo, CANADA

## Abstract

We explored the effect of gravity on the perceived representation of the absolute distance of objects to the observers within the range from 1.5–6 m. Experiments were performed on board the CNES Airbus Zero-G during parabolic flights eliciting repeated exposures to short periods of microgravity (0 g), hypergravity (1.8 g), and normal gravity (1 g). Two methods for obtaining estimates of perceived egocentric distance were used: verbal reports and visually directed motion toward a memorized visual target. For the latter method, because normal walking is not possible in 0 g, blindfolded subjects translated toward the visual target by pulling on a rope with their arms. The results showed that distance estimates using both verbal reports and blind pulling were significantly different between normal gravity, microgravity, and hypergravity. Compared to the 1 g measurements, the estimates of perceived distance using blind pulling were shorter for all distances in 1.8 g, whereas in 0 g they were longer for distances up to 4 m and shorter for distances beyond. These findings suggest that gravity plays a role in both the sensorimotor system and the perceptual/cognitive system for estimating egocentric distance.

## Introduction

Because of its constant presence throughout Earth’s history, terrestrial gravity (1 g) has influenced and shaped life. Testing individuals in altered gravity allows investigating its specific role in biological systems mechanisms such as those involved in visual space perception [1]. The visually perceived space refers to a perceptual representation of the immediate physical environment. A major goal of vision research is to characterize the mapping from physical to visual (perceived) space under different conditions of information availability.

To reach for an object, we need to know the absolute distance between this object and our body. The exact signals that are required by the central nervous system to calculate this perceived egocentric distance are still unknown. Visual cues, including binocular disparity, relative motion parallax, angular declination, aerial perspective, linear perspective, and texture gradients, are known to contribute to distance perception [2]. When we move our finger along an object surface, the haptic cues also help us to perceive the size and shape of the object, and therefore to determine its distance from our body [3]. Recent studies also suggest that vestibular information might contribute to the perception of visual space. For example, measurements of the perceived distance of visual targets were found to be dependent on head position and orientation relative to gravity [4–7]. Alterations in visual perception of objects size were also observed in healthy subjects in microgravity [8–10]. Because our perception of size depends closely on our perception of distance [2], our hypothesis was that perceived distance should also be altered in microgravity.

Parabolic flight is the only ground-based condition in which microgravity (0 g) can be created long enough for safely testing changes in human perception and behavior. In addition to the 0 g period, parabolic flight generates equal durations of 1.8 g, which present another unique opportunity to test the same responses to hypergravity and back in 1 g. The 0 g and 1 g conditions are characterized by alteration in sensory inputs from tactile, proprioceptive, and otolith receptors. By measuring the perceived distance in these conditions, our objective was to better understand the mechanisms that set the neurocognitive, vestibular and proprioceptive systems’ functioning and adaptation towards changing gravity levels.

A common method used for obtaining distance estimates is visually directed walking, or *blind walking*, in which the participants attempt to walk while blindfolded to the location of the previously viewed target [11–12]. In a typical experiment, the participants view a target on the ground and then are asked to close their eyes and walk to the target’s location. Because the participants’ eyes are closed, they must rely on proprioceptive and vestibular cues, as well as efferent copy, to update their body movement. Based on this sensorimotor information, participants are fairly accurate up 9 m. The distance is increasingly underestimated, however, as the distance further increases [13–14]. Besides blind walking, participants can also make verbal reports of perceived distance by using conventional metric representations of a foot, meter, or other unit of measure. The actual values of verbal report are generally smaller than those of walking distance [15].

**Fig 1:**
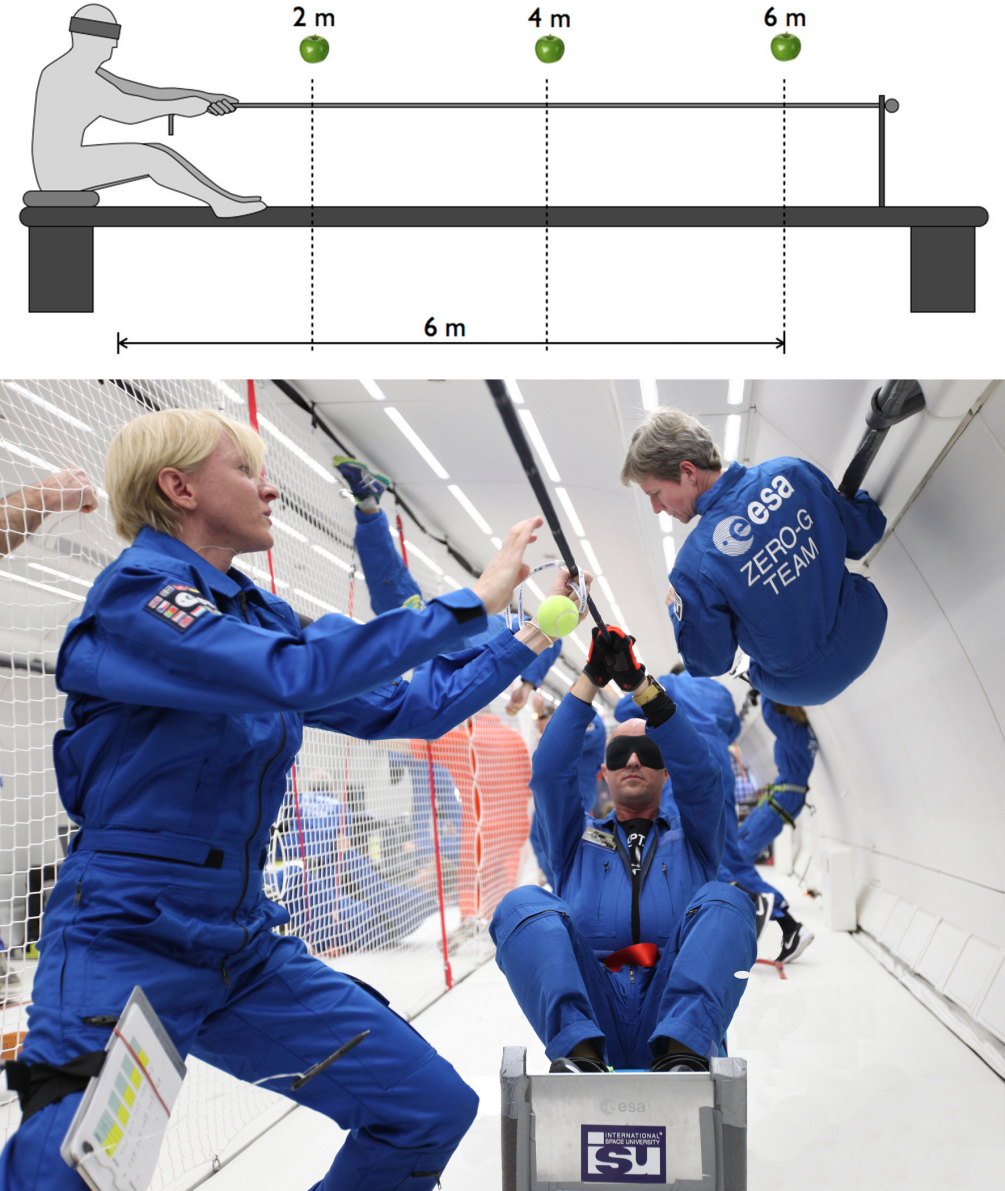
Principle of blind pulling. Top. Blindfolded subjects seated on a sled translated toward the memorized visual target by pulling on a rope attached to the end of the structure. Bottom. Photograph of the experiment in the Airbus A-310 Zero-G aircraft (photo courtesy of Novespace).

Verbal reports of perceived distance are easy to perform in 0 g and 1.8 g. However, walking in 1.8 g during parabolic flight is forbidden by the flight safety authorities because it poses a fall hazard. Also, subjects cannot walk in 0 g because in free-fall the subjects feet are no longer in contact with the aircraft floor. We have therefore developed a modified version of blind walking, which we called *blind pulling*, in which blindfolded subjects were seated in a sled and translate toward the memorized visual target by pulling on a rope with their arms (Fig 1). Just like blind walking, blind pulling is an open loop, visually directed action toward a memorized visual target, in which vestibular and proprioceptive cues also participate in the perceptual response. We therefore compared the subjects’ ability to accurately estimate distances during blind pulling in 0 g, 1.8 g, and 1 g during parabolic flight.

## Materials and Methods

### Ethics Statement

Nine subjects (3 females, 6 males), ranging in age from 21 to 59 years (mean 36.4 years) participated in this study. All subjects had passed the equivalent of an Air Force Class III medical examination, and had normal or corrected-to-normal vision with no known visual deficits. The experiment was undertaken with the understanding and written consent of each subject. The test procedures were approved by the European Space Agency medical board and by the Comité de Protection des Personnes Nord Ouest III (Caen, France) and were performed in accordance with the ethical standards laid down in the 1964 Declaration of Helsinki.

### Procedure for Blind Pulling

The equipment included a visual target (a tennis ball) that was positioned at 10 different distances between 1.5 and 6 m from the subject at eye level, and a sled mounted on a 7-m long rail attached to the aircraft floor. The subjects sat with their lap and feet strapped to the sled, and moved along the rail by pulling on a rope that was tensioned with turnbuckles to vertical beams attached at both ends of the rail above the subjects’ head (Fig 1). The rail was attached to the aircraft’s seat tracks, along the x-direction of the aircraft, facing forward or backward, using supporting beams and nut fittings. A uniform padding covered up the most salient markers (beams, bolts, windows) within the aircraft to minimize any information that could be used by the subjects to calculate the distance.

An experimental trial started the subject wearing a blindfold with his back against one of the vertical beam. An operator suspended a tennis ball on the rope at eye level at a predetermined distance. The subject removed the blindfold, looked at the tennis ball, put back the blindfold and pulled the rope to move the seat until she thought her nose was the closest to the tennis ball (in the meantime, the operator had removed the tennis ball). When the subject thought she had reached the target, she verbally reported the distance travelled (in m and cm). An operator noted both the verbal estimate and the final position of the subject and the actual position of the visual target. The subject then returned with the sled to the original position by hand-pulls. At no point during the test did the subjects receive a feedback on their distance estimate performance. A 3-axis accelerometer (Gulf Coast Data Concept, LLC, Waveland, MS, USA) was mounted on the sled to record both gravity level and sled motion. Two video cameras recorded the whole experiment.

### Experimental Protocol

The experiment took place during two ESA campaigns of parabolic flights (186 parabolas) on board the CNES Airbus Zero-G aircraft. Because no blind pulling data exist in the literature, it was necessary to compare the distance estimates during both blind pulling and blind walking with the same subjects. These tests were done days in advance of the flights in a stress-free condition comparable to the laboratory environment. One to three days prior to the parabolic flight, distances estimates were measured in 1 g during blind walking on the ground on an unmarked roadway. The same tennis ball as used in the experimental setup in the aircraft was placed on the road at various distances and the subjects were instructed to briefly look at the ball, close their eyes and walk to the ball. Distances estimates during blind walking were then compared to distance estimates during blind walking in the aircraft while it was parked on the runway. Verbal estimates of distances were also obtained in both preflight ground tests. Ten different distances between 1.5 and 6 m were tested three times each, randomized across subjects and trials.

During the flights, each subject was tested during 20 parabolas (10 different distances; two trials each) in 1 g, 0 g, and 1.8 g. The order of the visual target distances was randomized across gravity levels and across subjects. Each parabola started with a pull up phase at 1.8 g, followed by a free-fall phase (0 g), and ended with a pull out phase at 1.8 g, all lasting about 20 sec. Because the 1.8 g net acceleration was not exactly perpendicular to the aircraft floor (the aircraft had a 2–3 angle of attack, thus generating a pitch tilt of the resulting gravito-inertial acceleration) the subjects were facing the back of the aircraft during the first campaign and the front of the aircraft during the second campaign. Most subjects took prophylactic medication (a combination of promethazine and dexedrine) before boarding the plane, and none of them showed symptoms of motion sickness during the flight. Controls in 1 g were also performed on board the aircraft during straight and level flight between successive parabolas, while the medicated subjects were under the influence of the drug. This was to ensure that the changes seen across the various gravity levels were not due to the effect of the medication.

### Data Analysis

The distance estimates of each target were plotted as function of the true target distance. The distance traveled or reported was fitted by the output of Lappe et al.’s [16] leaky spatial integrator model. For the task of moving to a previously described distance, this model predicts perceived distance (x) at which the subject believed they had reached the target for a given target distance (d) according to the following equation:

x=−(1/a)*ln(k/(d*a+k))

where *k* is the sensory gain (k = 1 for an ideal observer) and *a* represents the leaky integrator constant or leak rate (a = 0 for an ideal observer) [17].

The distance perceived by each individual using blind walking, blind pulling and verbal reports were compared across target distance and conditions with repeated measures ANOVAs in Excel. Using an alpha error of 0.05 as the decision rule, the null hypothesis was that there is no difference across gravity level and target distance.

## Results

### Distance Estimates in 1 g on the Ground

**Fig 2:**
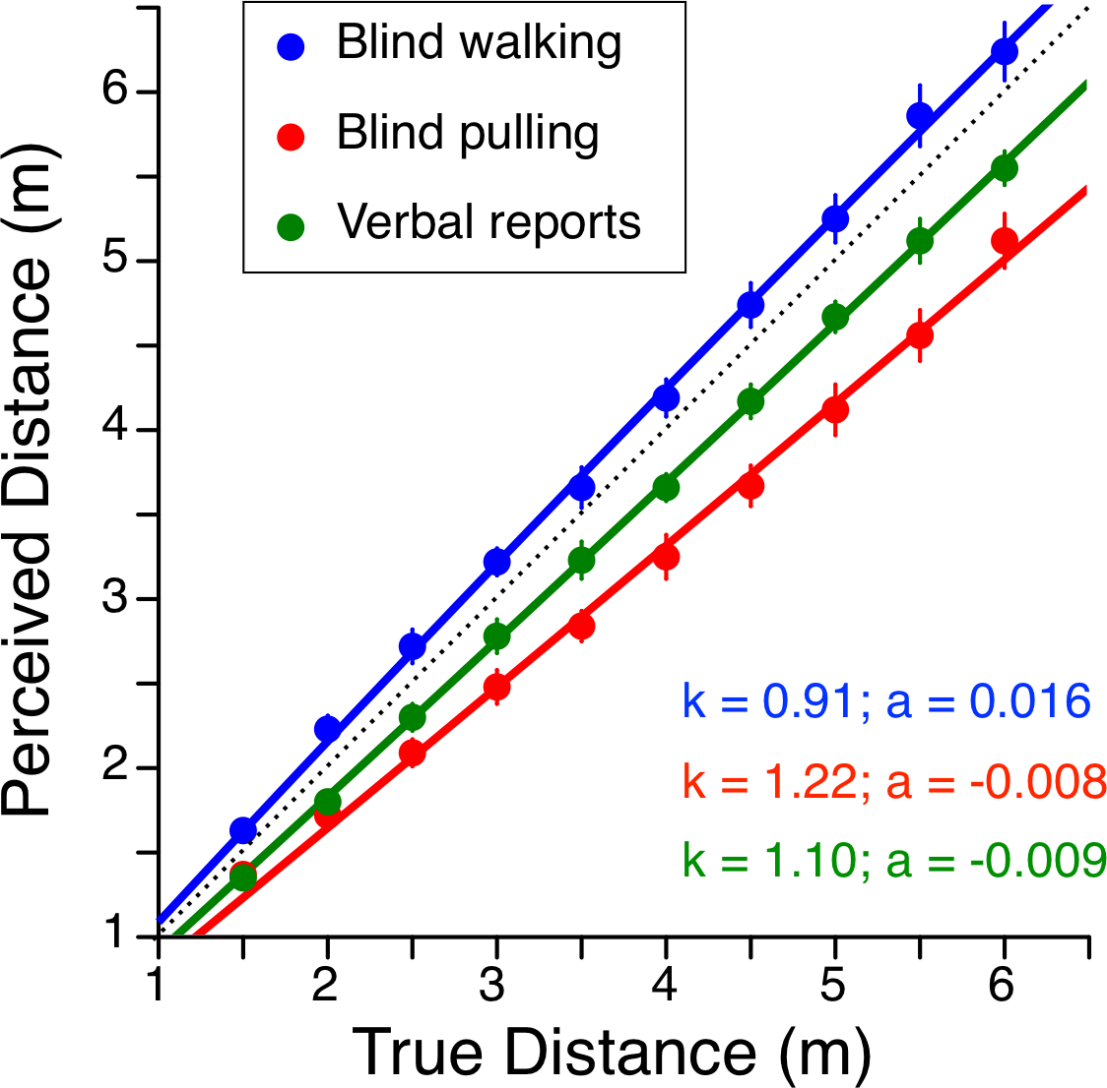
Preflight comparison between the perceived distances estimated using blind walking, blind pulling, and verbal reports for each target distance (true distance). Mean ± SE of 9 subjects. The continuous lines are the fits by the leaky integration model described in [16]. *k* is the gain and *a* is the spatial decay constant; the dashed line indicates veridical performance (*k* = 1; *a* = 0). Individual data are reported in S1 Table.

Verbal estimates of perceived distance were not significantly different [F (1, 160) = 3.7, p = 0.06] on the road (blind walking) and in the aircraft parked on the runway (blind pulling), so the results were averaged for both conditions. The individual distance estimates were then analyzed using a 10 (targets) x 3 (estimate methods; blind walking, blind pulling, verbal reports) repeated-measures two-way ANOVA, alpha = 0.05. There was a significant effect of target distance [F (9, 240) = 489, p < 0.001], a significant effect of estimate method [F (2, 240) = 148, p < 0.001], and a significant effect of interaction [F (18, 240) = 2.14, p < 0.001]. Using blind walking, the subjects overestimated the distance by 6.7% in average (SE 0.8%). On the contrary, when using verbal reports, distances were underestimated by 8.0% (SE 0.4%). During the blind-pulling tests, distances were even more underestimated (mean 16.2%; SE 1.1%) (Fig 2).

### Distance Estimates in 1 g, 0 g, and 1.8 g during Parabolic Flight

**Fig 3:**
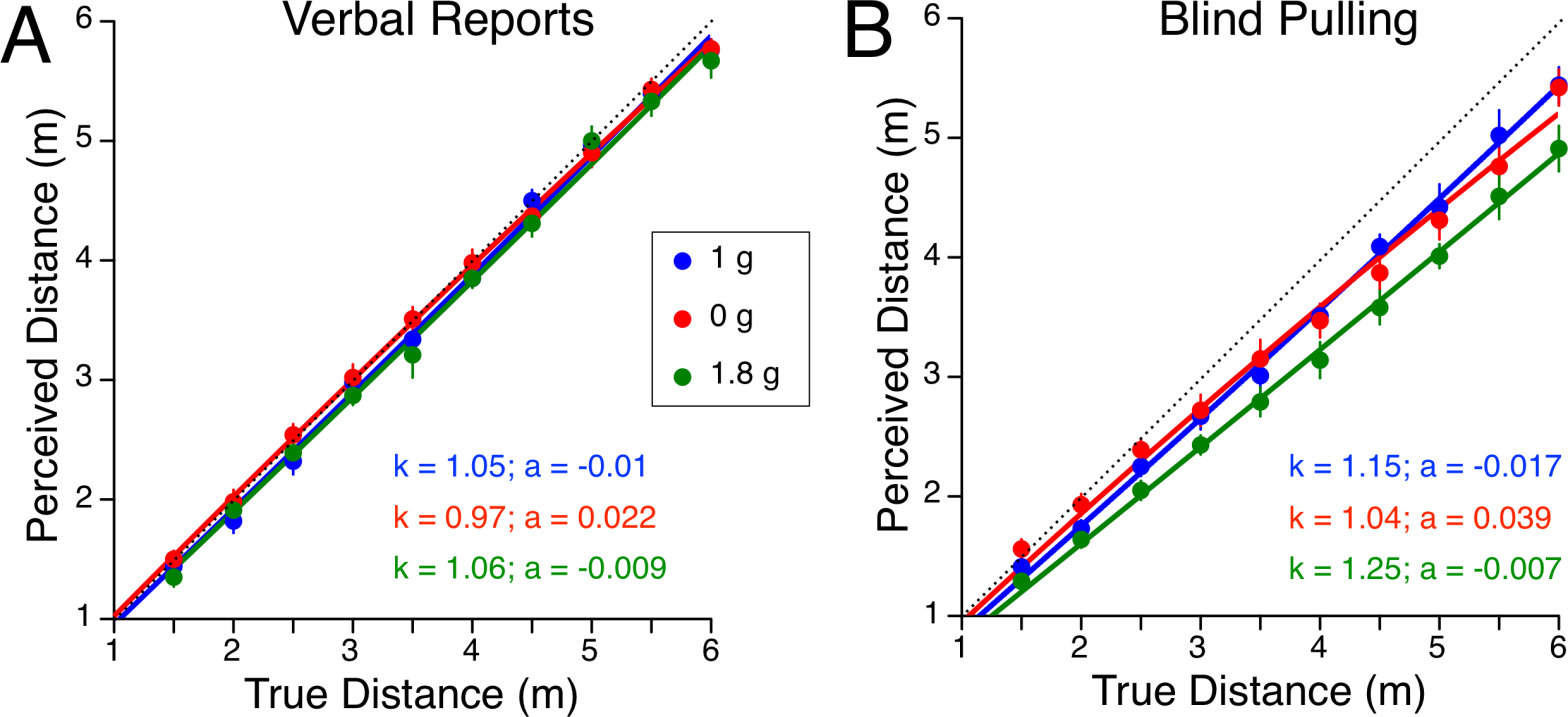
**Perceived distances for verbal reports (A) and blind pulling (B) for each target distance across the three gravitational levels during parabolic flight.** Mean ± SE of 9 subjects. Individual data are reported in S1 Table.

Verbal estimates of distances were not significantly different using the blind pulling apparatus on the ground and in the aircraft under level flight (1 g) conditions [F (1, 160) = 0.94, p = 0.335). However, blind pulling distance estimates were significantly different [F (1, 160) = 18.6, p < 0.001]. The Lappe model suggests less spatial decay during level flight than on the ground (Fig 3A).

The distance estimates taken during the flights using verbal reports and blind pulling were each analyzed using a 10 (targets) x 3 (gravity; 1 g, 0 g, 1.8 g) repeated-measures ANOVA. For verbal reports, there was a significant effect of target distance [F (9, 240) = 650, p < 0.001], and gravity level [F (2, 240) = 3.1, p < 0.05], but no significant effect of interaction [F (18, 240) = 0.54, p = 0.934]. For blind pulling, there was also a significant effect of target distance [F (9, 240) = 258, p < 0.001] and gravity level [F (2, 240) = 16.2, p < 0.001], but no significant effect of interaction [F (18, 240) = 0.57, p = 0.917] (Fig 3B).

**Fig 4:**
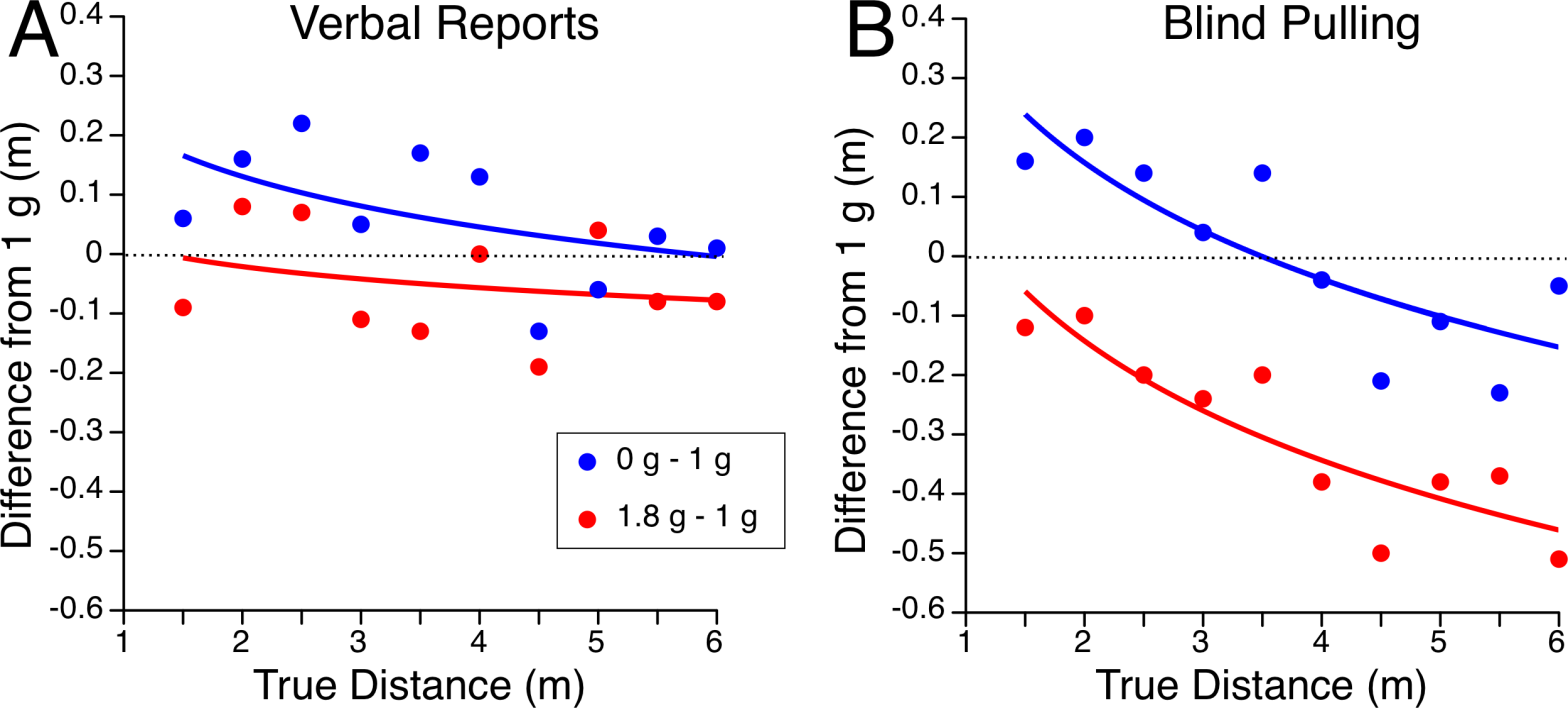
**Differences in perceived distances between the 1 g condition and microgravity (0 g– 1 g) and hypergravity (1.8 g– 1 g) for verbal reports (A) and blind pulling (B).** Individual data are reported in S1 Table.

The differences from the 1 g data for both the 0 g and 1.8 g conditions are plotted in Fig 4. These graphs show that there are clear differences in distance estimates between normal gravity, microgravity, and hypergravity.

**Fig 5:**
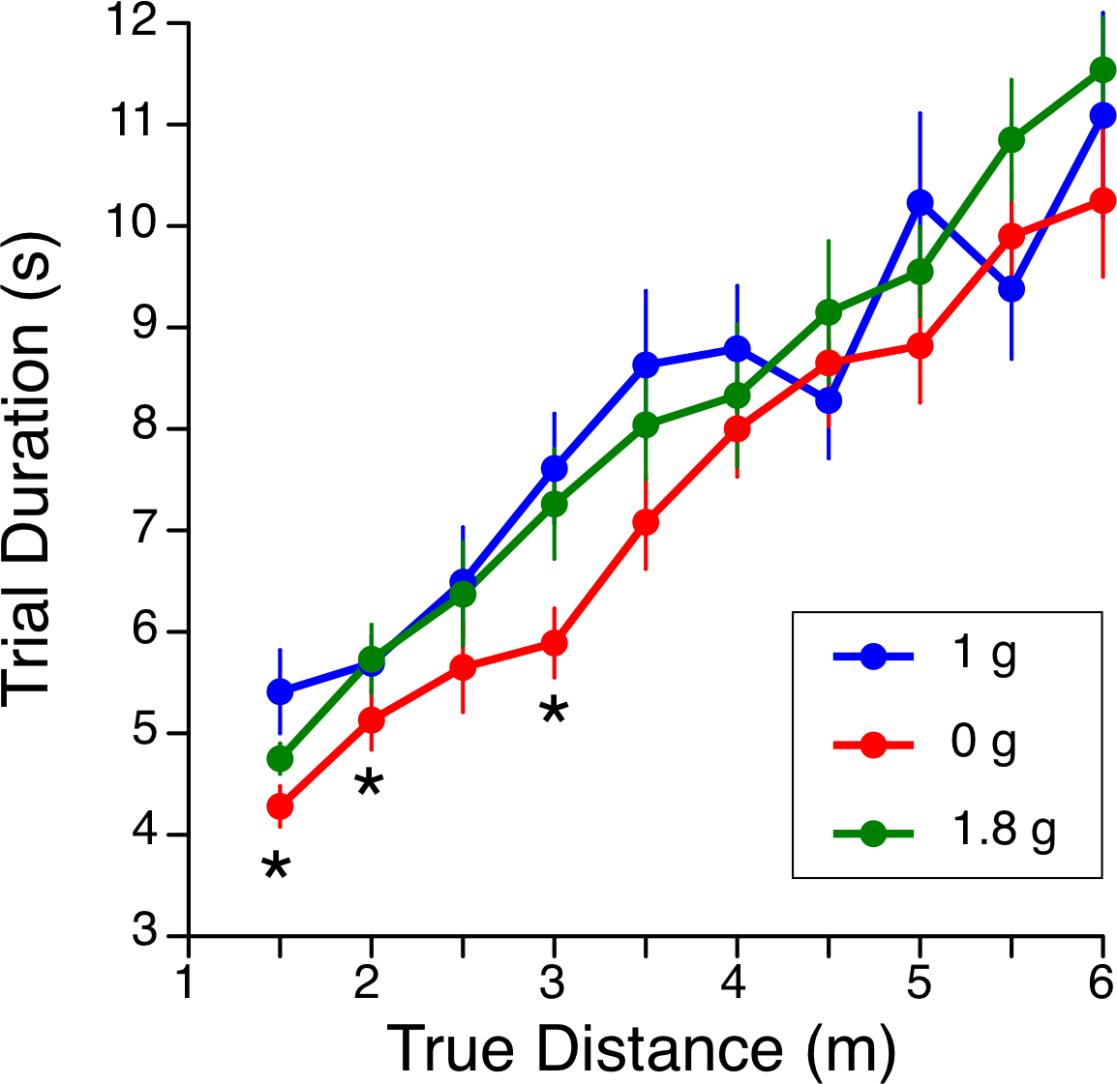
Comparison between the duration to perform the blind pulling task for each target distance in the three gravitational levels. Mean ± SE of 9 subjects; * p < 0.05 relative to 1 g. Individual data are reported in S1 Table.

The time for performing the blind pulling task was compared across the three gravity conditions to verify if the speed for accomplishing the test was altered by the gravity level. The duration of blind pulling trials was significantly longer as the distance increased [F (9, 240) = 37.5, p < 0.001] and there was a significant effect of the gravity level [F (2, 240) = 6.28, p = 0.002]. Paired t-tests indicated that the subjects were accomplishing the blind pulling task faster at 0 g than at 1 g for distances of 1.5 m, 2.5 m, and 3 m (Fig 5).

## Discussion

The results of this study indicate that the ability to estimate egocentric distance by direct motion is affected by the gravity level. Compared to normal gravity, distances are perceived to be shorter in hypergravity, and longer in microgravity at least for distances less than 4 m. Distance estimates using verbal reports are also affected by gravity, but at a lesser extent. This finding suggests that gravity plays a role in both the sensorimotor system (pulling) and in the perceptual/cognitive system (verbal) for perceiving the distance of objects.

### Distance Perception in 1 g

Our measurements of walked distance and verbal reports in 1 g are in agreement with those of Loomis and Philbeck [18]. Walked distance is highly accurate for target distances up to 9 m and shows undershooting for longer distances. This good accuracy has been taken as evidence that observers accurately perceive the target location as seen from their initial viewpoint [13–14]. This interpretation relies upon the assumption that locomotion is well calibrated, so that the distance observers walk accurately reflects their perceived target distance. Verbal reports, on the other hand, are less accurate [19]. In fact, verbal report and walked/pulled distance use cognitively quite different mechanisms. Both rely on an estimation of the distance between observer and object, but verbal report requires the conversion of the perceived distance in abstract symbolic reference, which may depend highly on previous experience. Walked/pulled distance on the other hand requires the conversion of perceived distance in a bodily enactment (perceived travelled distance similar to perceived egocentric distance).

Blind pulling in 1 g reveals a clear underestimation of pulled distance. One possible interpretation is that pulling is not as well calibrated as walking because we don't usually use the arm muscles to translate our body forward. The length of the arms is shorter than the legs, and consequently the distance between two pulls on the rope with the hands is shorter than a step with the feet. If subjects use the same number of arm pulls than the steps for travelling to the perceived distance, then they systematically underestimate this distance.

The differences in pulling distance estimates in 1 g during the flight compared to the tests performed on the runway might be related to the effects of vibrations, noise, stress [20] and/or motion sickness medication. However, these factors are present during all phases of the flight, so they do not confound the comparison of subject performance between the three gravity levels.

### Distance Perception in 0 g and 1.8 g

When subjects actually looked at the visual targets just prior to blind pulling, the ground surface provided an important frame of reference for estimating their distances [2]. One possible interpretation for why perceived distance was different in 0 g and 1.8 g is that eye height was misperceived due to the changes in otolith and proprioceptive cues. However, a recent study showed that free-floating astronauts on board the ISS continue to use the body eye height to estimate the width of apertures in microgravity [21]. This finding suggests that even when there is no contact with the floor, subjects continue to accurately estimate visual eye height.

Gaze position is another important factor for distance estimation [22]. Studies in parabolic flight have shown that gaze position actually shifted down in 0 g and up in 1.8 g [23]. In our blind pulling setup the visual targets were presented at eye level, so the angular declination was not informative. Nevertheless, some subjects may have perceived that the targets were not exactly in the straight-ahead direction, which could have influenced their distance estimates.

Another possibility is that the misperception of distance in altered gravity could be related to a misrepresentation of the apparent size of the environment. Geometrical visual illusions of object size are less present in 0 g [8–9], as well as when observers are tilted relative to the gravitational vertical [24]. Spaceflight studies also point out to potential changes in the astronauts’ perceived visual space. Illusions of self-position and motion, spatial disorientation, and perceptual limitations are commonly felt by astronauts in orbit. For example, the depth cues from linear perspective are less salient [25], and the perception of objects’ height and depth is different [26], perhaps as a consequence of a perceptual rescaling of space [27–28]. When the gravitational reference is altered, such as in microgravity or in hypergravity, the depth system may get recalibrated resulting in a rescaling of the apparent size of the environment with corresponding errors in size perception [7]. This interpretation is supported by recent brain imagery studies which have revealed that vestibular activation could indeed modulate the excitability of visual cortical regions [29],

When subjects were pulling with their arms along the sled while blindfold in altered gravity, distances misperception could occur because of (a) imperfect monitoring of command signals to the arm muscles; (b) incorrect scaling of afferent signals for muscle, joint and pressure receptors; (c) incorrect information from the otolith receptors signaling body translation; or (d) incorrect expectations of the relation between efferent and afferent signals. Changes in motor behavior due to an alteration of proprioceptive inputs have been previously observed in microgravity and hypergravity. During pointing with the arm, subjects tend to over-reach memorized visual targets in low gravity and to under-reach in high gravity and on return to normal gravity after spaceflight [30]. Distortions of weight and mass perception also occur when manipulating objects in altered gravity environments [31].

Another possible explanation is the “gravity theory” [32] and other effort-based theories [20], which suggest that perceived distance corresponds with anticipated muscular effort needed for locomotion. More effort is made in climbing slopes and staircases than in walking horizontally on the ground. If muscular effort is transformed into apparent distance, then vertical locomotion distance is perceived to be longer than the horizontal locomotion distance. According to these theories, the distances should appear farther in 1.8 g compared to 1 g as it takes more effort to move against gravity, whereas the distances should appear closer in 0 g because there is less friction. As a matter of fact our measurements indicate that subjects go a little faster in the 0 g condition. However, our distance perception data show that apparent distances are closer in 1.8 g and farther in 0 g, which is the opposite of what the gravity theory predicts. Other authors have shown distance perception results that are in contradiction with the gravity theory [7,33].

## Conclusions

Visual perception is of primary importance for spatial orientation and object recognition. Because the static vestibular (otolithic) and proprioceptive signals are altered in reduced gravity, astronauts become increasingly dependent on vision to perceive motion and orientation [1]. Misperception of distance could therefore be a risk for astronauts on the surface of the Moon or Mars [34–35]. The adaptation of humans to spaceflight needs to be analyzed to understand the underlying mechanisms that regulate human psycho-physiological adaptive process to changing gravity. Human psycho-physiological health has to be safeguarded and possibly improved when long-term human space missions are programmed in the near future.

The results of this experiment could also benefit the knowledge of human factors during spaceflight. Imagine the following contingency scenario during an activity outside the International Space Station (ISS): a failure of the spacesuit life support system causing fog inside the astronaut’ helmet visor that completely occludes the visual field. In fact, several Shuttle and ISS crewmembers have experienced this problem when the anti-fog system leaked within their spacesuit [36]. Before his visor is completely fogged, the astronaut would visually estimate the distance between himself and the airlock. He would then translate along the ISS rail system, in complete absence of vision, until he believes he has reached the airlock. The time spent to accomplish this homing navigation and its accuracy is critical because of the potential concomitant other failures in the spacesuit life support system. During training for this contingency scenario, the crewmember should be made aware that he might underestimate the distance travelled along the rail while in 0 g.

## Supporting Information

S1 Table Individual data set obtained for all conditions and analysis of variance. (XLS)

## References

[1] Clément G, Reschke MF. Neuroscience in Space New York: Springer; 2008.

[2] Sedgwick HA. Space perception. In: Boff KR, Kaufman L, Thomas JP, editors. Handbook of Perception and Human Performance: Vol. 1. Sensory Processes and Perception. New York: Wiley; 1986. pp 21.1–21.57.

[3] Battaglia PW, Kersten D, Schrater PR. How haptic size sensations improve distance perception. PLoS Comput Biol. 2011;7(6): e1002080 10.1371/journal.pcbi.1002080 21738457 PMC3127804

[4] Wu B, He ZJ, Ooi TL. (2007). The linear perspective information in ground surface representation and distance judgment. Percept Psych. 2007;69: 654–672. 10.3758/bf03193769 17929690

[5] Higashiyama A, Adachi K (2006). Perceived size and perceived distance of targets viewed from between the legs: Evidence for proprioceptive theory. Vis Res. 2006;46: 3961–3976. 16979687 10.1016/j.visres.2006.04.002

[6] Toskovic O. Brave upside down world–Does looking between the legs elongate or shorten the perceived distance? Psichologija. 2010;43: 21–31.

[7] Harris L, Mander C. Perceived distance depends on the orientation of both the body and the visual environment. J Vision. 2014;14: 17. 10.1167/14.12.17 25319945

[8] Villard E, Tintó Garcia-Moreno F, Peter N, Clément G. Geometric visual illusions in microgravity during parabolic flight. NeuroReport. 2005;16: 1395–1398. 16056146 10.1097/01.wnr.0000174060.34274.3e

[9] Clément G, Richard G, Lockerd A, Lathan C. Geometric illusions in astronauts during long-duration spaceflight. NeuroReport. 2012;23: 894–899. 10.1097/WNR.0b013e3283594705 22955144

[10] Clément G, Skinner A, Lathan C. Distance and size perception in astronauts during long-duration spaceflight. Life (Basel). 2013;3: 524–537. 25369884 10.3390/life3040524 PMC4187133

[11] Thomson JA. How do we use visual information to control locomotion? Trends Neurosci. 1980;3: 247–250.

[12] Thomson JA. Is continuous visual monitoring necessary in visually guided locomotion? J Exp Psychol: Hum Percept Perf. 1983;9: 427–443. 10.1037//0096-1523.9.3.427 6223981

[13] Loomis JM, Da Silva JA, Philbeck JW, Fukusima SS. Visual perception of location and distance. Cur Dir Psychol Sci. 1966;5: 72–77.

[14] Philbeck JW, Loomis JM. A comparison of two indicators of perceived egocentric distance under full‐cue and reduced cue conditions. J Exp Psychol: Hum Percept Perf. 1997;23: 72–85. 10.1037//0096-1523.23.1.72 9090147

[15] Loomis JM, Da Silva JA, Fujita N, Fukusima SS. Visual space perception and visually directed action. J Exp Psychol: Hum Percept Perf. 1992;18: 906–921. 10.1037//0096-1523.18.4.906 1431754

[16] Lappe M, Jenkin M, Harris LR. Travel distance estimation from visual motion by leaky path integration. Exp Brain Res. 2007;180: 35–48. 17221221 10.1007/s00221-006-0835-6

[17] Harris LR, Herpers R, Jenkin M, Allison RS, Jenkin H, Kapralos B, et al The relative contributions of radial and laminar optic flow to the perception of linear self-motion. J Vision. 2012;12: 7. 10.1167/12.10.7 22976397

[18] Loomis JM, Philbeck JW. Measuring spatial perception with spatial updating and action In: Klatzky RL, Behrmann B, MacWhinney B, editors. Embodiment, Ego-Space, and Action. New York: Psychology Press; 2008 pp 1–43.

[19] Andre J, Rogers S. Using verbal and blind-walking distance estimates to investigate the two visual systems hypothesis. Percept Psychophys. 2006;68: 353–361. 16900829 10.3758/bf03193682

[20] Proffitt DR, Stefanucci J, Banton T, Epstein W. The role of effort in perceiving distance. Psychol Science. 2003;14: 106–112. 10.1111/1467-9280.t01-1-01427 12661670

[21] Bourrelly A, McIntyre J, Luyat M. Perception of affordances during long-term exposure to weightlessness in the International Space station. Cognitive Processing 2015;16 (Suppl 1): 171–174. 10.1007/s10339-015-0692-y 26224263

[22] Gajewski DA, Wallin CP, Philbeck JW. Gaze behavior and the perception of egocentric distance. J Vision. 2014;14: 20. 10.1167/14.1.20 PMC3900371 24453346

[23] Clément G, André-Deshays C, Lathan CE. Effects of gravitoinertial force variations on vertical gaze direction during oculomotor reflexes and visual fixation. Aviat Space Environ Med. 1989;60: 1194–1198. 2604675

[24] Clément G, Eckardt J. Influence of the gravitational vertical on geometric visual illusions. Acta Astronaut. 2005;56: 911–917. 15835042 10.1016/j.actaastro.2005.01.017

[25] Lathan C, Wang Z, Clément G. Changes in the vertical size of a three-dimensional object drawn in weightlessness by astronauts. Neurosci Lett. 2000;295: 37–40. 11078931 10.1016/s0304-3940(00)01584-6

[26] Clément G, Allaway HCM, Demel M, Golemis A, Kindrat AN, Melinyshyn AN, et al Long-duration spaceflight increases depth ambiguity of reversible perspective figures. PLoS One 2015;10(7): e0132317 10.1371/journal.pone.0132317 26146839 PMC4492703

[27] Stefanucci JK, Geuss MN. Big people, little world: The body influences size perception. Perception. 2009;38: 1782–1795. 20192128 10.1068/p6437 PMC3298356

[28] Van der Hoort B, Guterstam A, Ehrsson HH. Being Barbie: The size of one’s own body determines the perceived size of the world. PloS One. 2011;6(5): e20195 10.1371/journal.pone.0020195 21633503 PMC3102093

[29] Seemungal BM, Guzman-Lopez J, Arshad Q, Schultz SR, Walsh V, Yousif N. Vestibular activation differentially modulates human early visual cortex and V5/MT excitability and response entropy. Cerebral Cortex. 2013;23: 12–19. 10.1093/cercor/bhr366 22291031 PMC3513948

[30] Lackner JR, DiZio P. Motor function in microgravity: Movement in weightlessness. Cur Opin Neurobiol. 1996;6: 744–750. 10.1016/s0959-4388(96)80023-7 9000028

[31] Clément G. Effects of varying gravity levels in parabolic flight on the size-mass illusion. PLoS One. 2014;9(6): e99188 10.1371/journal.pone.0099188 24901519 PMC4047103

[32] Howard IP, Templeton WB. Human spatial orientation New York: Wiley; 1996.

[33] Higashiyama A. Horizontal and vertical distance perception: The discorded-orientation theory. Percept Psychophys. 1996;58: 259–270. 8838168

[34] Daum SO, Hecht H. Distance estimation in vista space. Attent Percept Psychophys. 2009; 71: 1127–1137. 10.3758/APP.71.5.1127 19525542

[35] Oravetz CT, Young LR, Liu AM. Slope, distance and height estimation of lunar and lunar-like terrain in a virtual reality environment. Gravit Space Biol. 2009;22: 57–66.

[36] NASA. Significant Incidents and Close Calls in Human Spaceflight: EVA Operations. Houston: NASA JSC Flight Safety Office, JS-2011-010; 2011.

